# Visual Function and Disability Are Associated With Focal Thickness Reduction of the Ganglion Cell-Inner Plexiform Layer in Patients With Multiple Sclerosis

**DOI:** 10.1167/iovs.18-25809

**Published:** 2019-03

**Authors:** Ce Shi, Hong Jiang, Giovana Rosa Gameiro, Huiling Hu, Jeffrey Hernandez, Silvia Delgado, Jianhua Wang

**Affiliations:** 1 School of Ophthalmology and Optometry, Wenzhou Medical University, Wenzhou, Zhejiang, China; 2 Department of Ophthalmology, Bascom Palmer Eye Institute, University of Miami Miller School of Medicine, Miami, Florida, United States; 3 Department of Neurology, University of Miami Miller School of Medicine, Miami, Florida, United States; 4 Shenzhen Key Laboratory of Ophthalmology, Shenzhen Eye Hospital, Jinan University, Shenzhen, China

**Keywords:** tomographic thickness, ganglion cell-inner plexiform layer, multiple sclerosis, low-contrast visual acuity, disability

## Abstract

**Purpose:**

The purpose of this study was to visualize the topographic thickness patterns of the intraretinal layers and their associations with clinical manifestations in patients with multiple sclerosis (MS).

**Methods:**

Ninety-four eyes of 47 relapsing-remitting MS patients without history of optic neuritis were imaged using optical coherence tomography and compared with 134 eyes of 67 healthy subjects. Volumetric data centered on the fovea were segmented to obtain the thickness maps of six intraretinal layers. The thickness measurements partitioned using the Early Treatment Diabetic Retinopathy Study (ETDRS) partition were correlated to the Expanded Disability State Scale (EDSS) and Sloan low contrast visual acuity (LCVA). The receiver-operating characteristics (ROC) curves were calculated to obtain the area under the ROC curves (AUCs).

**Results:**

The ganglion cell-inner plexiform layer (GCIPL) showed horseshoe-like thickness reduction profoundly at the nasal sector. The most profound thickness reduction zone (circular area, diameter = 1 mm) was located at 2 mm in the nasal sector and 0.4 mm inferior from the fovea, named the “M zone.” The thickness reduction of the M zone was −7.3 μm in MS eyes, which was the most profound alteration, compared to any ETDRS sectors. The AUC of the M zone was 0.75. The relationship between the thickness of the M zone and EDSS (*r* = −0.59, *P* < 0.001) or 2.5% LCVA (*r* = 0.51, *P* < 0.001) were ranked as the strongest relation compared to any ETDRS sectors.

**Conclusions:**

This is the first study, to our knowledge, to visualize focal thickness alteration of GCIPL and reveal its relationship to visual function and disability in patients with MS without history of optic neuritis.

Multiple sclerosis (MS) is a chronic autoimmune-mediated inflammatory disease, featured as neurodegeneration in the central nervous system.^1^ Current available MS treatments generally target the autoimmune and inflammatory aspects of MS and vary in effectiveness across patients.^2^ Demyelinating lesion burden and brain atrophy measured by conventional magnetic resonance image (MRI) remain a standard measure of clinical trial outcomes. However, MRI is time-consuming, expensive, and impacted by various other factors such as hydration status and age. Hence, non-MRI techniques to assess the neurodegeneration of MS in vivo are needed in order to monitor the efficacy of new clinical interventions and disease progression.

The involvement of the visual pathway in patients with MS has been well documented, and the retina containing unmyelinated nerve fibers provides a window for studying neurodegeneration.^3^,^4^ With the advent of optical coherence tomography (OCT), the thickness of the peripapillary retinal nerve fiber layer (pRNFL) containing unmyelinated axons and of the ganglion cell layer-inner plexiform layer (GCIPL) containing retinal ganglion cell bodies can be readily measured in vivo. Thinning of the pRNFL and GCIPL is associated with MS-related visual dysfunction,^5^ disability,^6^ and brain atrophy.^7^,^8^ Thus, these retinal neurodegenerative changes are regarded as an imaging biomarker of global central nervous system degeneration in MS.^8^–^10^ Although these measurable OCT variables are used to monitor the efficacy of clinical trials,^11^,^12^ they are often based on the averaged thickness of these retinal layers, which may compromise their sensitivity for detecting subtle changes. Indeed, alterations of the average thickness of pRNFL and GCIPL in longitudinal follow up of patients with MS are often below 1 μm yearly,^11^ and current approaches do not have adequate sensitivity to discern changes in thickness at this scale, impeding the ability to monitor treatment efficacy in clinical trials.^11^

A further confounding factor is that the thickness of intraretinal layers is not evenly distributed,^13^,^14^ and the alterations of each intraretinal layer could be different due to various disease mechanisms.^15^ Current advancements in OCT and its robust ability to segment intraretinal layers enable detailed topographic analysis and visualization of intraretinal layer thickness, which may reveal disease-specific imaging biomarkers of MS.^14^,^16^ Furthermore, the localized changes in quadrants and sectors may help us to better understand the underlying disease mechanism, which could be used to differentiate MS retinal changes from other neurodegenerative diseases (such as glaucoma and age-related neurodegeneration).^15^ For example, when compared to the average pRNFL, the temporal pRNFL (pRNFL-T) thickness was found to be the most relevant surrogate measure for both physical and cognitive disability in relapsing-remitting MS (RRMS),^17^,^18^ indicating that detailed partition may help establish a more robust association between the imaging markers and clinical findings.

We hypothesize that detailed analyses of the topographic thickness of intraretinal layers can identify the most vulnerable locations of neurodegeneration that are primarily associated with visual function and disability in patients with MS. The goal of the present study was to visualize the topographic thickness patterns of the intraretinal layers and their associations with clinical manifestations in patients with MS.

## Methods

### Participants

The study was approved by the research review board of the University of Miami and conducted in accordance with the Declaration of Helsinki. All subjects signed informed consent forms. All patients with MS who were seen at the MS Center of Excellence at the University of Miami were asked about their interest in participating in the present study. The patients who expressed interest were then scheduled study visits with a neuro-ophthalmologist (HJ) at the Bascom Palmer Eye Institute. Eligible patients were recruited from the neuro-ophthalmology clinic at the Bascom Palmer Eye Institute, University of Miami. Patients with MS were diagnosed in the MS Center of Excellence of the Department of Neurology, the University of Miami, and the diagnosis of MS was made based on the 2010 revised McDonald Criteria.^19^ Physical disability was assessed according to the expanded disability status scale (EDSS). A total of 47 RRMS patients without history of optic neuritis were recruited. The patients' disease activity during the last 2 years before the study enrollment or since the first clinical episode (if the disease duration was shorter than 2 years) were recorded. The disease activity included the number of patients with relapsed or increased T2 lesions or gadolinium-enhancing (Gd+) lesions as shown on a brain MRI. The patients who were free of relapses and Gd+ lesions and who showed stable T2 lesion counts and EDSS values within this period were defined as having no evidence of disease activity (NEDA-3). The patients' disease-modifying therapies (DMTs) included glatiramer acetate, interferon beta-1a/b, teriflunomide, or dimethyl fumarate, and the second-line DMT included natalizumab, fingolimod, or rituximab (Table 1). Fingolimod is a structural analog of sphingosine-1-phosphate and has been used as an oral immunomodulating agent for RRMS. Macular edema was an infrequent side effect, and the images obtained from the HD-OCT (Cirrus HD-OCT; Carl Zeiss Meditec, Dublin, CA, USA) of the macula were inspected for possible edema in patients on fingolimod.^20^ The controls were recruited from people who came for annual eye exams. Subjects with other systemic or ocular diseases or refractive errors of more than ±6 diopters were excluded.

**Table 1:** Demographic Characteristics of Study Subjects

	**RRMS**	**Healthy Controls (HCs)**	***P*** **Value**
Subjects	47	67	
Age, mean ± SD, y	41.1 ± 10	38.1 ± 10.8	0.08
Sex, F:M	40:7	48:19	0.09
Duration, y	6.7 ± 5.6		
Patients (%)
Disease activity 2 y before enrollment			
Relapses	7 (14%)		
New T2 lesions	8 (17%)		
Gd+ lesions	8 (17%)		
DMT			
None	11 (23%)		
DMT	36 (77%)		
Mean (standard deviation)
LCVA (1.25%)	27.6 ± 8.9		
LCVA (2.5%)	51.0 ± 7.1		
EDSS	2.0 ±1.7, IQR (1.00–2.75)		

M, male; F, female; LCVA, low-contrast sensitivity visual acuity; IQR, interquartile range.

Each patient with MS had an ophthalmic screening, including intraocular pressure and slit-lamp examination. High-contrast visual acuity was tested using Snellen charts. All patients with MS had best corrected visual acuity equal to or above 20/20 in both eyes, which was confirmed by subsequent chart review. Binocular testing of low-contrast visual acuity (LCVA) was done with both the 1.25% and 2.5% low-contrast Sloan letter acuity chart (Sloan chart, Precision Vision, LaSalle, IL, USA) on a retro-illuminated cabinet at 2-m distance, with the best possible correction for refractive errors. LCVA scores were recorded as the number of letters correctly read by the patient.^21^

In addition, the Cirrus HD-OCT was used to acquire GCIPL and peripapillary RNFL (pRNFL) thickness using the 512 × 128 scan protocol.^22^,^23^ The measurements of GCIPL with the Zeiss elliptical partition and quadrantal pRNFL were obtained from the Cirrus HD-OCT reports. As the raw datasets of the Cirrus HD-OCT device were not accessible, no detailed segmentation of the Zeiss OCT datasets was performed in the present study. Furthermore, the ultra-high-resolution (UHR)-OCT dataset analyzed (see below) did not yield the elliptical partition, making it impossible to compare the two OCT devices used in the present study.

### Intraretinal Topographic Thicknesses Measured Using UHR-OCT

**Figure 1:**
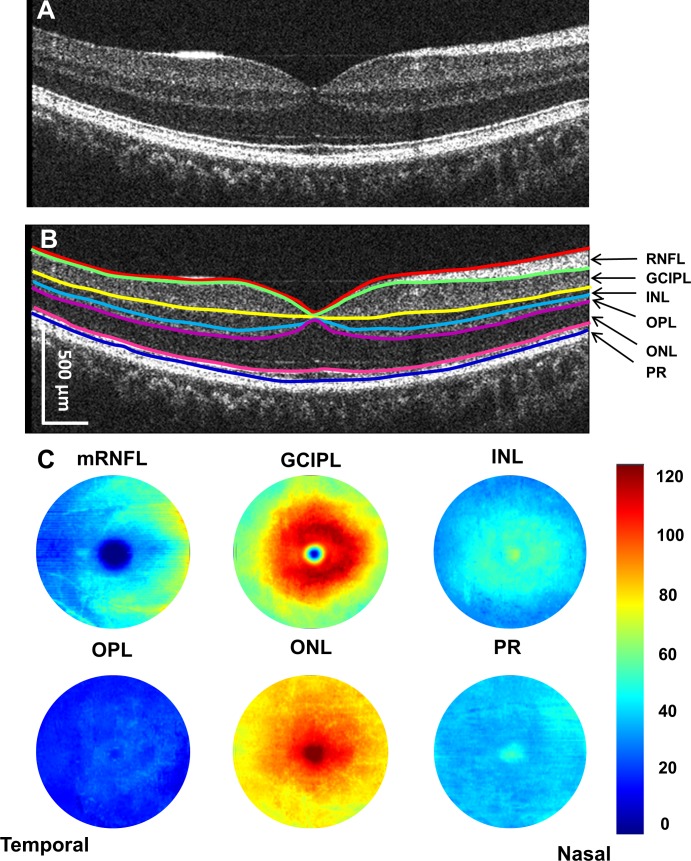
Cross-sectional retina and segmented topographic thickness maps of intraretinal layers. Using UHR-OCT, the retina of a healthy subject was imaged (A). Six layers of intraretinal layers were segmented (B). Seven boundaries defined these six intraretinal layers and created six topographic maps (with a diameter of 6 mm) (C). mRNFL, macular retinal nerve fiber layer; INL, inner nuclear layer; ONL, outer nuclear layer; OPL, outer plexiform layer; PR, retinal photoreceptor. The scale bar in (B) is in nominal scale.

**Figure 2:**
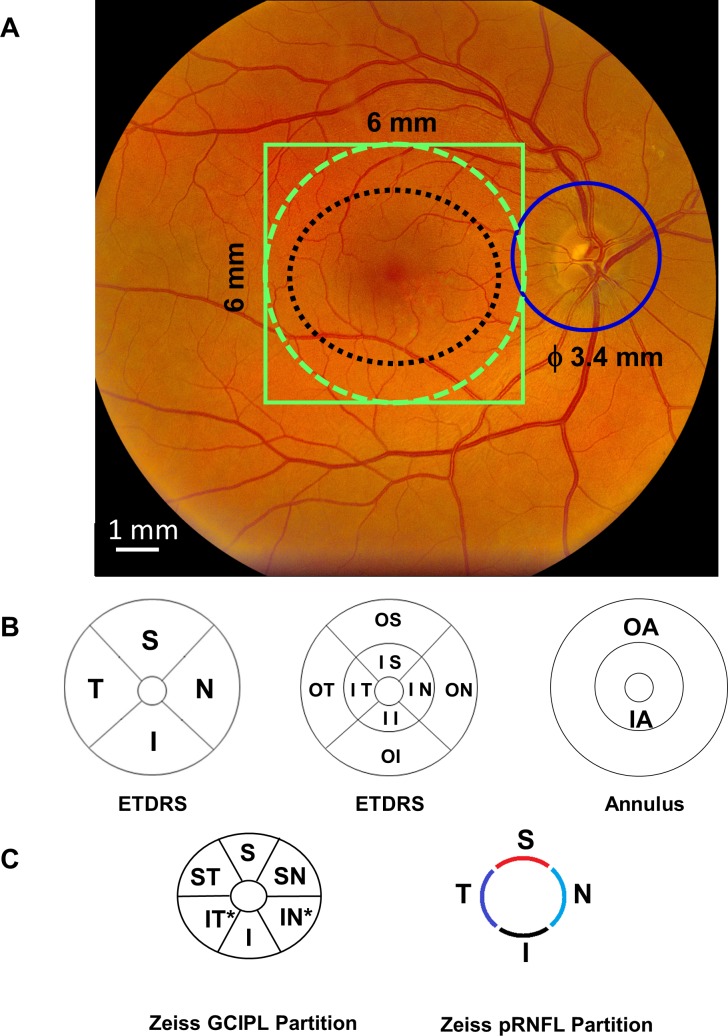
OCT imaging protocols and partition methods for analyzing the topographic thickness of the retina. The 6 × 6-mm scan protocol was used to scan the macula centered on the fovea using UHR-OCT (solid green line) and Zeiss HD-OCT (black dotted line) (A). The retina with a diameter of 6 mm (green dotted line) was segmented using the Orion software to obtain six intraretinal thickness maps. In addition, a 6 × 6-mm scan protocol was used to scan the optic nerve head to obtain the pRNFL scan with a diameter of 3.4 mm (solid blue circle) (A). The partition methods were used to define sectors of the intraretinal layers for calculating the topographic thickness. UHR-OCT measured the thickness within a 27.48 mm^2^ annulus (from 1 to 6 mm in diameter). In the ETDRS definition, the quadrantal division was performed using 45° and 135° medians (B). The central 1-mm zone of the fovea was removed. In addition, three concentric rings with diameters of 1, 3, and 6 mm were used to divide the map into nine zones (B). The inner and outer annuli were also divided (B). Using Zeiss elliptical partition, six sectors were divided from the dataset scanned using the Zeiss HD-OCT (C). In addition, the pRNFL scan using the Zeiss HD-OCT was divided into four quadrants (C). S indicates superior; I, inferior; T, temporal; N, nasal; IS, inner superior; II, inner inferior; IT, inner temporal; IN, inner nasal; OS, outer superior; OI, outer inferior; OT, outer temporal; ON, outer nasal. In Zeiss elliptical partition, ST indicates superior temporal; SN, superior nasal; IN*, inferior nasal; IT*, inferior temporal.

The custom UHR-OCT device has been described in previous reports.^14^,^24^ Briefly, the system was a spectral-domain OCT with an axial resolution of ∼3 μm (in tissue) and a scan speed of 24,000 A-scans per second. An area of 6 × 6 mm centered on the fovea was acquired using a 512 × 128 pixels macular cube protocol with 128 consecutive B scans and 512 A-scans per B scan, which constitutes a three-dimensional volume. Automatic image-processing software (Orion; Voxeleron LLC, Pleasanton, CA, USA) was used to process the database to segment six intraretinal layers (Fig. 1). Orion software is a commercially available OCT analysis program for research use only and can be used with the datasets acquired using commercial OCT devices such as Zeiss Cirrus HD-OCT (with special data exportation)^23^ and Spectralis OCT.^25^ This software had been successfully applied in previous publications by us^14^,^16^,^24^ and others.^23^,^25^ The segmented intraretinal layers were RNFL, GCIPL, inner nuclear layer (INL), outer nuclear layer (ONL), outer plexiform layer (OPL), and retinal photoreceptor (PR), in addition to the total retinal thickness (TRT). Segmented thickness maps were visually inspected, and all segmentation was successfully accomplished in all eyes without any correction. The Early Treatment Diabetic Retinopathy Study (ETDRS) partition was used to further analyze the thickness maps (Fig. 2). Additionally, all data of the thickness and coordinates of the center of fovea were exported for generating thickness maps. The center of the fovea was used to align the dataset, then the thickness of each of the 512 × 128 pixels was averaged in the group, creating the average thickness map of each intraretinal layer.^16^ Thickness differentiation maps were created by subtracting the average thickness of the control group from that of the MS group. Both eyes of each study subject were imaged.^16^

### Statistical Analyses

All data were analyzed with statistical software (SPSS, ver. 25; Statistical Package for the Social Sciences, IBM, Armonk, NY, USA). Continuous variables are presented as the mean ± standard deviation. Analysis of covariance (ANCOVA) was used to analyze topographic thicknesses in sectors and annuli between groups, with the adjustment of age and sex. An ANOVA post hoc test was used to compare the difference between different sectors. Partial correlation adjusting for age and sex was used to determine the relation between the structural parameters and clinical magnifications. *P* < 0.05 was considered statistically significant. Thickness maps were rendered using software (Matlab, ver. 2014b; Mathworks, Natick, MA, USA).

## Results

Demographics and baseline characteristics are listed in Table 1. Eight patients were taking fingolimod, and none of them had maculopathy based on Cirrus HD-OCT macular images. Nine patients had disease activity within 2 years, and there were no significant differences in thickness measurements in these patients compared to patients without disease activity (all *P* > 0.05). There were no significant differences of any thickness measurements in patients with MS with and without DMTs (all *P* > 0.05). Results are presented as the mean ± standard deviation.

### Intraretinal Thickness Maps

**Figure 3:**
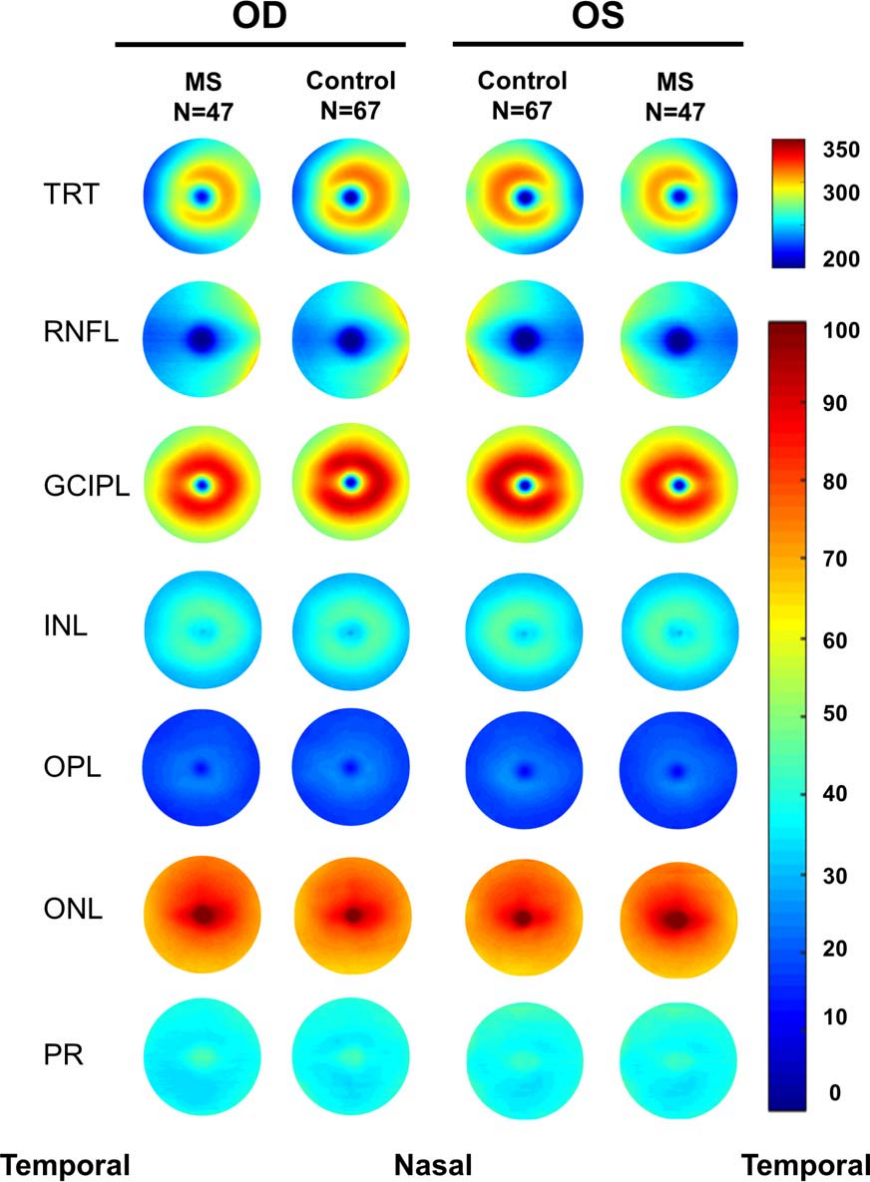
Intraretinal thickness maps. Aligned with the foveal center, the thickness maps of six intraretinal layers obtained using UHR-OCT were averaged in each group of the MS and control groups, showing the difference in thickness among two groups in both right and left eyes. Note the symmetry of the thickness maps between the right and left eyes. mRNFL, macular retinal nerve fiber layer. Scale bar unit: micrometers.

**Figure 4:**
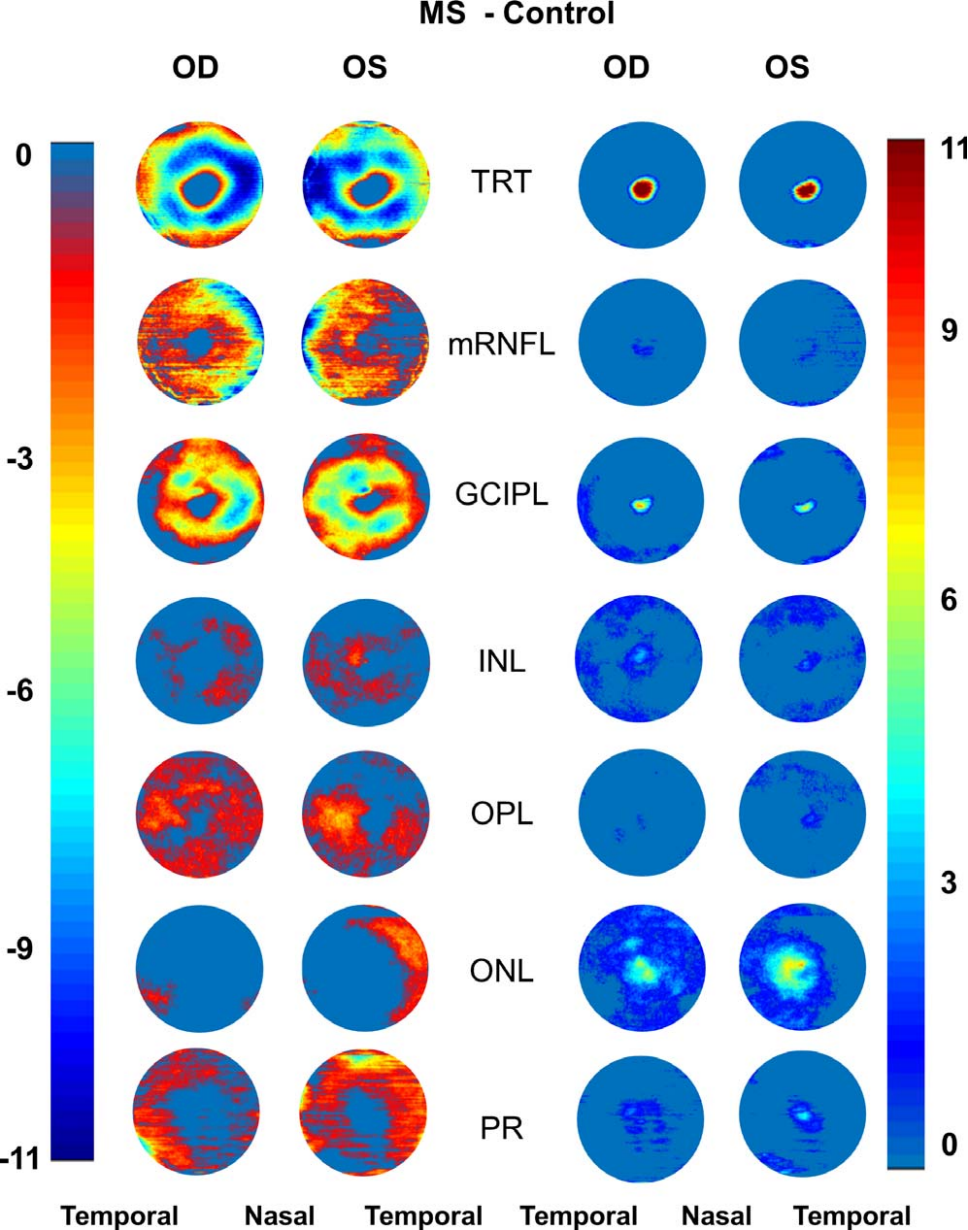
Negative and positive alterations of intraretinal thickness maps. Subtracting the thickness maps obtained using UHR-OCT of the control group from that of the MS group, the negative (reduced thicknesses) and positive (increased thicknesses) alterations were visualized. In the MS group, negative changes (reduced thicknesses) mainly happened in TRT, mRNFL, GCIPL, OPL, and PR layers in both eyes. Increased thicknesses were evident in the ONL. Scale bar unit: micrometers.

Aligned using the center of the fovea, which was detected by the Orion software, the thickness map of each intraretinal layer was averaged in each group of the MS and control groups (Fig. 3). The symmetric patterns of the intraretinal thickness maps were evident between the right and left eyes in both groups. However, the average maps showed the difference in the thickness maps between the MS and control groups. By subtracting the thickness map of the control group from the MS group, the changes in the intraretinal layer thickness were visualized in the MS group (Fig. 4).

### Focal Thickness Changes of the GCIPL

**Figure 5:**
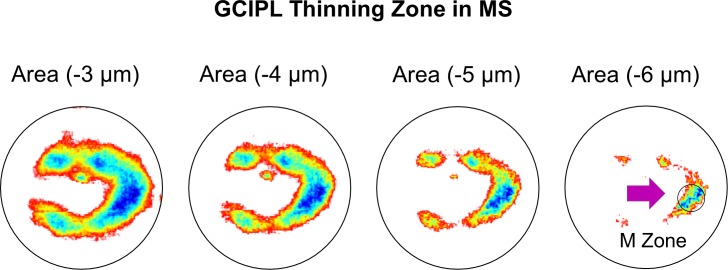
Focal thickness alteration of the GCIPL in MS eyes. Using different values of the thickness changes, the areas of focal thickness reduction were visualized in MS eyes compared to the control eyes. The most profound thinning zone appeared to locate at nasal 1.98 mm and inferior 0.42 mm from the fovea with a circle zone (diameter = 1 mm), the MS thinning zone of the GCIPL (M zone). The average reduction of the M zone was −7.3 μm in MS eyes, which was higher than any GCIPL sectors using ETDRS partitions (P < 0.05), except for the IS sector (P = 0.08).

Using increasing values of the thickness changes, the areas of focal thickness changes were visualized in MS eyes relative to the control eyes. With the increasing cut-off values (3–4 μm), the thickness alteration pattern in MS eyes first showed focal thickness alteration in a horseshoe-like area around the fovea, which then focused on the island-like area at the nasal side at the cut-off value of 6 μm (Fig. 5).

**Figure 6:**
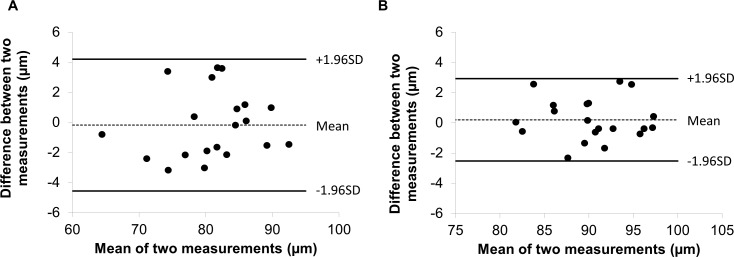
Bland-Altman plot for the M zone in the eyes of patients with RRMS and HCs. The Bland-Altman plot shows the agreement of the thickness measurements of the M zone was obtained twice in 20 eyes of patients with RRMS (A) and in 20 eyes of HCs (B).

The most profound thickness alteration zone appeared to locate at nasal 1.98 mm and inferior 0.42 mm from the fovea with a circular area (diameter = 1 mm), which was named the “M zone,” short for the MS thinning zone of the GCIPL. The GCIPL thickness of the M zone was 79.3 ± 9.3 μm in patients with MS, which was significantly less than that in healthy controls (HCs; 86.6 ± 5.7 μm, *P* < 0.001). The average thickness alteration of the M zone was −7.3 μm in the MS eyes, which was significantly higher than any GCIPL sectors (*P* < 0.05), except for the inner superior sector (*P* = 0.08) using the ETDRS partition. The thickness alteration of the M zone was higher than the sector inner temporal (IT*) (*P* = 0.02) and sector I (*P* = 0.02) of the elliptical partition, but was not different from the rest of sectors (superior nasal [SN]: *P* = 0.55; S: *P* = 0.20; inner nasal [IN*]: *P* = 0.27; and superior temporal [ST]: *P* = 0.08). In a subgroup containing 20 MS eyes and 20 control eyes, the M zone was measured twice in the same session on the same day. The coefficients of repeatability (calculated as 1.96 × SD of the differences) of the M zone were 4.0 μm in 20 MS eyes and 3.7 μm in 20 control eyes (Fig. 6). The intraclass coefficients of correlation of the M zone were 0.98 in 20 MS eyes and 0.99 in 20 control eyes, which were similar to the GCIPL in the other segmented region reported previously.^14^

**Figure 7:**
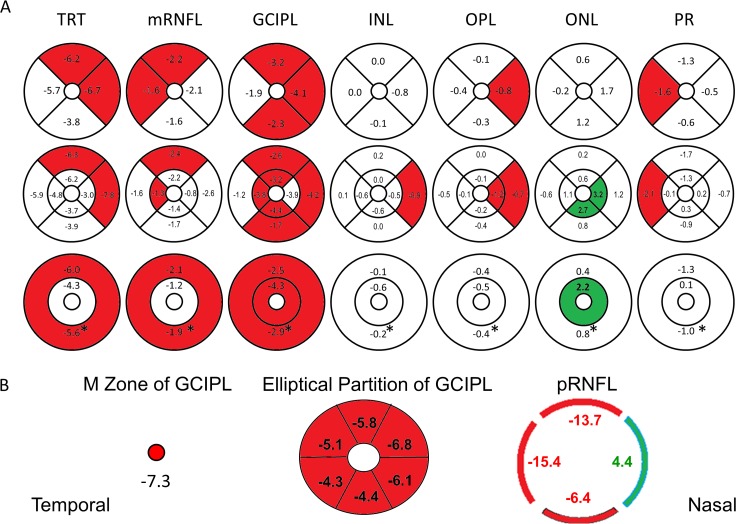
Thickness alterations of the intraretinal layers in patients with MS compared to controls. Significant thickness reduction (marked in red) was found in quadrantal, sectorial, and annular partitions (marked in red, P < 0.05) of all intraretinal layers, except for the ONL (A). The ONL showed significant thickness increase (marked in green) in the inner annulus (IA) and inner and outer nasal sectors. The GCIPL showed thickness reduction in the M zone, all sectors of the Zeiss elliptical partition, and the pRNFL showed thickness reduction in all quadrants, except for the nasal quadrant (thickening, marked in green) (B). *Annular thickness.

Using ANCOVA analysis, TRT, macular RNFL (mRNFL), and GCIPL in patients with MS showed significant decreases in thickness (Fig. 7; Tables 2–5), while analyses of quadrants (Table 2) revealed that the mRNFL showed significant thickness reduction in superior (S) and temporal (T) quadrants. The GCIPL showed significant thickness reduction in all quadrants except the temporal quadrant. Analyses of eight sectors using the ETDRS partition in the inner annulus (IA) (1–3 mm in diameter) and outer annulus (OA) (3–6 mm in diameter) showed significant thickness reduction of the mRNFL of the outer superior (OS) (*P* = 0.02) and IT (*P* = 0.04) sectors. Significant thickness reduction was also seen in all sectors of the GCIPL except for the IN (*P* = 0.07) and outer temporal (OT) (*P* = 0.36) sectors (Table 3). Analyses also showed significant thickness reduction in the annulus (*P* = 0.03) and the OA (*P* = 0.03) of the mRNFL (Table 4), and also in both inner (*P* = 0.02) and the outer (*P* = 0.01) annuli of the GCIPL. The changes in the ETDRS partition of the INL, OPL, ONL, and PR are listed in Figure 7. In addition to the thickness reduction of the GCIPL in the M zone, the GCIPL showed significant thickness reduction in all sectors of the Zeiss elliptical partition (all *P* < 0.001; Table 5). The pRNFL showed significant thickness reduction in the superior (*P* < 0.001; Table 5), temporal (*P* < 0.001), and inferior quadrants (*P* = 0.03), but also revealed significant thickness increase in the nasal quadrant (*P* = 0.03).

**Table 2:** Quadrantal Thicknesses of Intraretinal Layers in Patients With RRMS and HCs

**Layers**	**Group**	**Thickness, μm**			
		**I**	**N**	**S**	**T**
RNFL	RRMS	36.8 ± 4.2	41.8 ± 6.2	36.9 ± 4.8	24.6 ± 2.3
	HCs	38.4 ± 4.3	44.0 ± 6.2	39.1 ± 4.0	26.1 ± 3.1
	*P*	0.149	0.156	0.023	0.028
GCIPL	RRMS	66.6 ± 5.8	69.8 ± 6.1	66.1 ± 5.9	65.7 ± 5.2
	HCs	68.9 ± 4.6	73.9 ± 5.9	69.4 ± 4.5	67.5 ± 5.3
	*P*	0.013	0.001	0.006	0.145
INL	RRMS	35.7 ± 2.3	37.8 ± 2.3	36.9 ± 2.1	36.5 ± 2.7
	HCs	38.6 ± 2.5	38.6 ± 2.5	36.9 ± 2.1	36.6 ± 2.4
	*P*	0.524	0.081	0.987	0.668
OPL	RRMS	18.8 ± 3.6	19.1 ± 2.1	19.2 ± 2.3	19.4 ± 3.7
	HCs	19.1 ± 2.3	20.0 ± 3.0	19.3 ± 1.8	19.8 ± 2.9
	*P*	0.395	0.029	0.688	0.469
ONL	RRMS	72.9 ± 4.9	78.3 ± 5.4	78.4 ± 6.7	75.1 ± 5.7
	HCs	71.7 ± 3.8	76.6 ± 4.9	77.8 ± 4.3	75.3 ± 4.0
	*P*	0.395	0.029	0.688	0.469
PR	RRMS	35.6 ± 4.7	37.4 ± 4.1	37.7 ± 4.5	35.8 ± 4.9
	HCs	36.2 ± 4.1	37.9 ± 3.7	39.0 ± 5.9	37.4 ± 4.7
	*P*	0.596	0.489	0.109	0.040
TRT	RRMS	266.3 ± 13.3	284.1 ± 16.7	275.1 ± 13.9	257.1 ± 13.4
	HCs	270.1 ± 10.9	290.8 ± 15.3	281.3 ± 12.9	262.7 ± 12.2
	*P*	0.131	0.044	0.027	0.064

P values for the comparison between two groups by ANCOVA. I, inferior; N, nasal; S, superior; T, temporal.

**Table 3:** Sectoral Thicknesses of Intraretinal Layers in Patients With RRMS and HCs

**Layers**	**Group**	**Thickness, μm**							
		**II**	**IN**	**IS**	**IT**	**OI**	**ON**	**OS**	**OT**
mRNFL	RRMS	28.1 ± 3.3	24.8 ± 3.1	28.1 ± 4.2	21.8 ± 4.2	39.5 ± 4.7	46.9 ± 7.3	39.5 ± 5.2	25.4 ± 2.4
	HCs	29.5 ± 3.8	25.6 ± 2.8	29.5 ± 3.4	23.1 ± 2.0	41.2 ± 4.9	49.5 ± 7.5	41.9 ± 4.5	27.0 ± 3.8
	*P*	0.238	0.484	0.182	0.038	0.152	0.145	0.020	0.050
GCIPL	RRMS	82.4 ± 7.7	81.0 ± 8.9	82.2 ± 8.3	76.6 ± 7.0	61.7 ± 6.6	66.4 ± 5.9	61.4 ± 6.4	62.4 ± 5.5
	HCs	86.8 ± 7.5	84.9 ± 7.8	87.3 ± 7.4	80.4 ± 6.0	63.4 ± 5.7	70.6 ± 6.8	64.0 ± 4.7	63.6 ± 5.9
	*P*	0.016	0.073	0.007	0.017	0.049	0.001	0.024	0.363
INL	RRMS	42.8 ± 2.8	43.2 ± 3.1	43.5 ± 2.8	41.1 ± 2.8	33.5 ± 2.8	36.1 ± 2.4	34.9 ± 2.3	35.1 ± 2.9
	HCs	43.4 ± 3.4	43.7 ± 3.4	44.1 ± 3.0	41.7 ± 2.9	33.5 ± 2.5	37.0 ± 2.8	34.7 ± 2.3	35.0 ± 2.4
	*P*	0.572	0.730	0.593	0.591	0.579	0.047	0.820	0.469
OPL	RRMS	23.2 ± 7.2	22.4 ± 3.5	21.7 ±4.0	22.5 ± 5.5	17.4 ± 2.6	18.1 ± 1.8	18.4 ± 2.0	18.5 ± 3.3
	HCs	23.5 ± 3.4	23.6 ± 4.2	22.1 ± 3.0	22.6 ± 4.2	17.7 ± 2.3	18.9 ± 2.7	18.4 ± 1.5	19.0 ± 2.9
	*P*	0.718	0.045	0.591	0.730	0.255	0.036	0.748	0.393
ONL	RRMS	81.0 ± 7.0	88.3 ± 7.0	85.2 ± 6.7	84.6 ± 6.5	70.5 ± 4.6	75.3 ± 5.4	76.3 ± 7.3	72.2± 5.9
	HCs	78.3 ± 6.0	85.1 ± 6.9	83.5 ± 5.8	83.5 ± 5.5	69.7 ± 3.5	74.1 ± 4.6	76.1 ± 4.1	72.8 ± 3.8
	*P*	0.027	0.021	0.167	0.250	0.292	0.223	0.930	0.655
PR	RRMS	35.2 ± 4.5	37.4 ± 4.9	36.3 ± 5.3	36.4 ± 5.0	35.7 ± 5.2	37.4 ± 4.0	38.1 ± 4.5	35.6 ± 5.0
	HCs	34.9 ± 4.7	37.2 ± 4.7	36.3 ± 4.9	36.5 ± 4.7	36.6 ± 4.5	38.0 ± 3.9	39.8 ± 7.0	37.7 ± 5.0
	*P*	0.853	0.737	0.661	0.564	0.578	0.448	0.077	0.018
TRT	RRMS	292.7 ± 16.8	297.1 ± 18.7	297.0 ± 17.8	283.0 ± 16.7	258.3 ± 13.5	280.2 ± 16.7	268.6 ± 13.5	249.3 ± 13.2
	HCs	296.4 ± 16.1	300.1 ± 15.9	302.9 ± 15.7	287.8 ± 13.9	262.1 ± 12.0	288.0 ± 16.4	274.9 ± 14.0	255.1 ± 12.9
	*P*	0.490	0.554	0.168	0.219	0.102	0.020	0.021	0.060

P values for the comparison between two groups by ANCOVA. II, inner inferior.

**Table 4:** Annular Thicknesses of Intraretinal Layers in Patients With RRMS and HCs

**Layers**	**Group**	**Thickness, μm**		
		**IA**	**OA**	**Annulus**
RNFL	RRMS	25.7 ± 3.1	37.8 ± 4.3	35.0 ± 3.9
	HCs	26.9 ± 2.6	39.9 ± 3.9	36.9 ± 3.3
	*P*	0.141	0.031	0.034
GCIPL	RRMS	80.5 ± 7.6	63.0 ± 5.3	67.0 ± 5.2
	HCs	84.8 ± 6.8	65.4 ± 5.0	69.9 ± 4.5
	*P*	0.015	0.012	0.005
INL	RRMS	42.7 ± 2.7	34.9 ± 2.2	36.7 ± 2.2
	HCs	43.2 ± 3.0	35.1 ± 2.2	36.9 ± 2.1
	*P*	0.594	0.622	0.576
OPL	RRMS	22.5 ± 4.4	18.1 ± 2.1	19.1 ± 2.6
	HCs	23.0 ± 3.1	18.5 ± 2.1	19.5 ± 2.2
	*P*	0.379	0.184	0.225
ONL	RRMS	84.8 ± 6.1	73.6 ± 5.2	76.2 ± 5.1
	HCs	82.6 ± 5.2	73.2 ± 3.5	75.4 ± 3.7
	*P*	0.044	0.611	0.325
PR	RRMS	36.3 ± 4.7	36.7 ± 4.2	36.6 ± 4.1
	HCs	36.2 ± 4.4	38.0 ± 3.9	37.6 ± 3.7
	*P*	0.679	0.079	0.135
TRT	RRMS	292.5 ± 17.0	264.1 ± 13.2	270.7 ± 13.6
	HCs	296.8 ± 14.7	270.1 ± 11.9	276.3 ± 11.6
	*P*	0.316	0.023	0.040

P values for the comparison between two groups by ANCOVA.

**Table 5:** Thickness of the GCIPL and pRNFL in Patients With RRMS and HCs

**Layers**	**Regions**	**RRMS**	**HCs**	***P*** **Value**
GCIPL	S	78.9 ± 6.9	84.6 ± 5.4	<0.001
	SN	79.5 ± 8.1	86.3 ± 6.1	<0.001
	IN*	78.6 ± 7.8	84.6 ± 6.2	<0.001
	I	77.5 ± 5.6	81.9 ± 5.6	0.001
	IT*	79.1 ± 6.1	83.5 ± 5.4	0.001
	ST	77.6 ± 6.0	82.7 ± 5.3	<0.001
	Average	78.5 ± 6.7	83.9 ± 5.3	<0.001
pRNFL	S	110.2 ± 16.9	123.9 ± 15.5	<0.001
	N	73.5 ± 9.1	69.1 ± 11.5	0.029
	I	122.5 ± 15.2	128.8 ± 18.6	0.030
	T	58.0 ± 10.7	73.4 ± 14.4	<0.001
	Average	91.1 ± 9.5	98.8 ± 10.2	<0.001

P values for the comparison between two groups by ANCOVA.

### Discrimination Power

**Figure 8:**
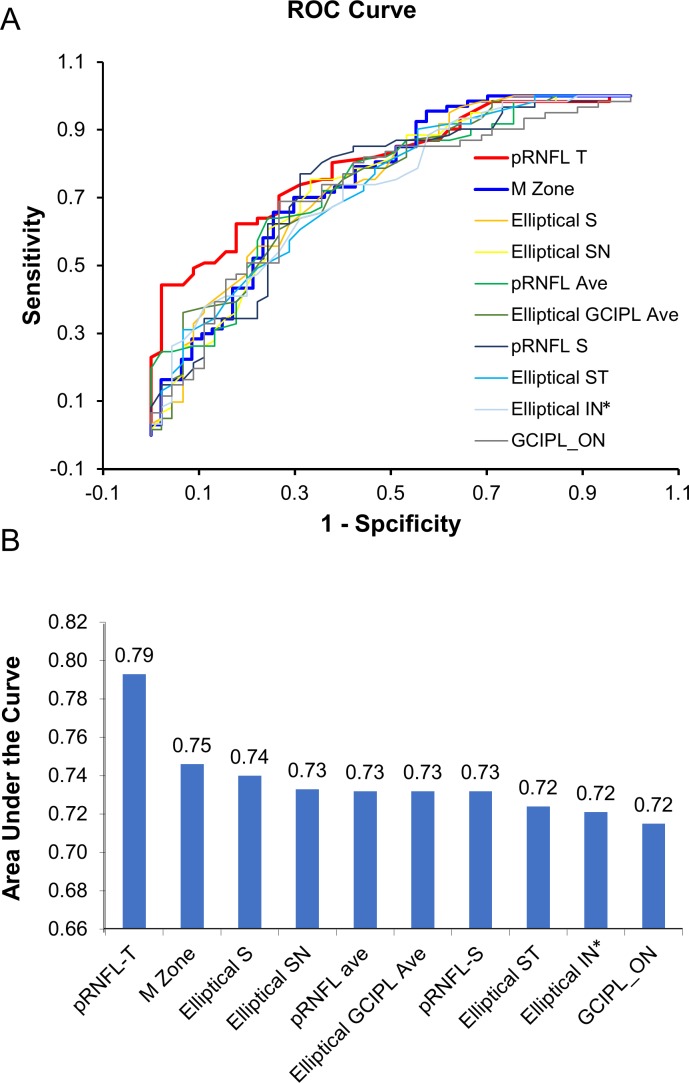
The discrimination power. The ROC curves (A) show that the value of AUCs of the M zone in MS eyes was 0.75, which was ranked second to pRNFL-T (B). The measurements between the right and left eyes were averaged.

The receiver-operating characteristics (ROC) curves showed that the area under the ROC curves (AUCs) of the M zone in MS eyes was 0.75, which was ranked second to pRNFL-T (Fig. 8). The M zone discriminated the patients with MS from the HCs at the sensitivity of 70.2% and specificity of 70.2%, with a cut of value of 83.9 μm.

### Correlations Between Different Partitions of the Intraretinal Layers With Clinical Parameters

**Figure 9:**
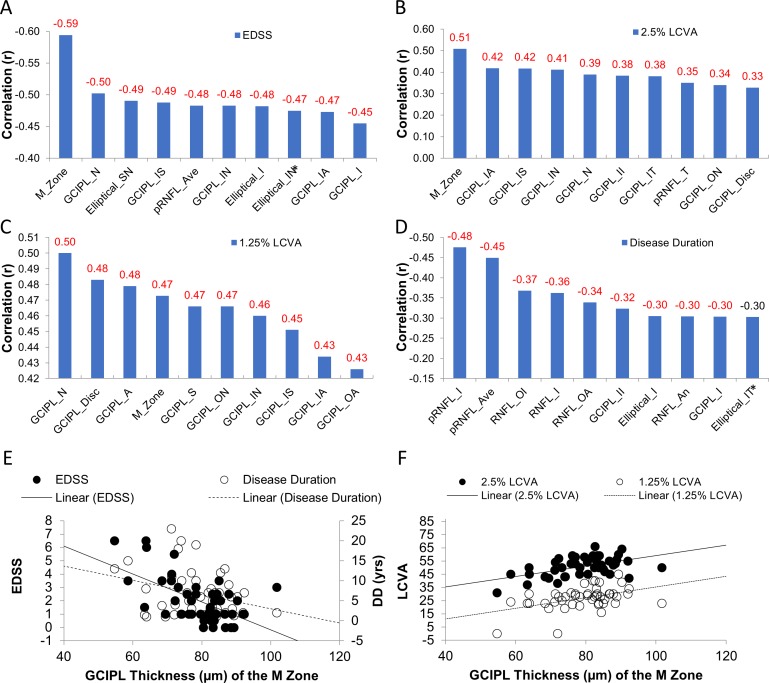
Correlations between the thicknesses of different partitions of the intraretinal layers with clinical parameters. The thickness of the M zone was negatively related to EDSS (r = −0.59, P < 0.001) (A), which was stronger than any other ETDRS sectors by ranking. The thickness of the M zone was also positively related to the 2.5% LCVA (r = 0.51, P < 0.001 (B), which was the strongest relation by ranking compared to any other ETDRS sectors. The thickness of the M zone was also positively related to 1.25% LCVA (r = 0.47, P < 0.001) (C), which was similar to the relation between GCIPL thickness in the nasal quadrant and 1.25% LCVA (r = 0.50, P < 0.001). The pRNFL-T was most strongly related to disease duration (D). The scatter plots (E, F) showed the relations between the thickness of the M zone and clinical manifestations. A, annulus; ave, average; II, inner inferior. Significant relationships are marked in red (P < 0.05). The measurements between the right and left eyes were averaged.

Using partial correlations, the thickness of the M zone was negatively related to EDSS (*r* = −0.59, *P* < 0.001; Fig. 9), which was ranked the strongest relation. The thickness of the M zone also was positively related to the 2.5% LCVA (*r* = 0.51, *P* = 0.001), which was the strongest relation by ranking compared to any other sectors. Finally, the thickness of the M zone was positively related to 1.25% LCVA (*r* = 0.47, *P* < 0.001), which was similar to the relation between GCIPL thickness in the nasal quadrant and 1.25% LCVA (*r* = 0.50, *P* < 0.001).

## Discussion

To our knowledge, this is the first study to analyze the various intraretinal layer thickness maps and to identify the most vulnerable location of GCIPL that was most strongly related to visual function and disability in patients with RRMS, without history of optic neuritis. According to our hypothesis, visualization and detailed analysis of intraretinal layer thickness help define the most profound focal changes of the intraretinal layers and establish the relationship between the most relevant focal alteration and clinical manifestations. Different partition methods and image protocols may reveal divergent sensitivities in detecting retinal neurodegeneration and associating that with clinical manifestations.^5^,^18^,^26^,^27^ However, the average thickness of pRNFL and GCIPL in MS patients was used in previous studies, often measured by commercial OCT devices (axial resolution of 5 μm) using conventional partitions such as the elliptical partition.^5^,^18^,^26^,^27^ Although 6- to 7-μm differences in the thickness of the pRNFL and GCIPL between MS without optic neuritis and control groups can be easily determined,^12^ the annual alteration in the average thickness (ranged from 0.2 to 1.3 μm per year) may not be easily detected in MS longitudinal clinical trials.^11^ In order to further develop the most sensitive imaging biomarkers to uncover the subtle changes in topographic thickness patterns, visualization of the intraretinal thickness maps and detailed partitions may be beneficial for clinical trials. A similar approach looking into regional thickness changes in the GCIPL and mRNFL has been successfully applied to other neurodegenerative diseases such as glaucoma^28^,^29^ and patients with Alzheimer's disease.^16^ Based on our results in the present study, the most profound thickness alteration was located on the nasal side of the GCIPL (M zone), which had the highest discrimination power compared to the other partitioned thickness measures in the macula, and provided both high sensitivity and specificity for differentiating focal thickness alteration in patients with MS from a normal population. The thickness of the M zone was also strongly related to LCVA and EDSS compared to other partitioned sectors, indicating that the parameter may be versatile and MS specific, which appears to be a potential disease-specific imaging biomarker.

Various patterns of retinal ganglion cell loss exist in different neurodegenerative disorders.^15^,^16^,^29^–^32^ The focal thickness alteration of the GCIPL (i.e., the M zone at the nasal region) in patients with MS is different compared to that in patients with glaucoma^29^ and Alzheimer's disease.^16^ The focal thinning of GCIPL is reported to be at the inferior temporal region (∼2 mm from the fovea) in patients with glaucoma^29^ and at the superior region in patients with Alzheimer's disease.^16^ The location of the M zone in patients with MS may indicate the preferential involvement of the papillomacular bundle in the parvocellular pathway. Indeed, pathologic studies showed that the smaller axons were damaged in patients with MS.^33^,^34^ Furthermore, mitochondrial dysfunction is considered to contribute to MS pathophysiology,^35^–^37^ and the location of the M zone is similar to the findings of mitochondrial optic neuropathies.^31^,^38^ In longitudinal studies of Leber's hereditary optic neuropathy, the nasal focal thinning of GCIPL occurred at the presymptomatic stage before the diffuse thinning of GCIPL.^30^,^31^ While the evidence from the present study supports that the M zone found in patients with MS could be developed as a more sensitive image marker, further longitudinal studies may validate whether the M zone can provide indicative information of disease progression and therapeutic efficacy.

In contrast to the M zone, other commonly used partition methods such as the ETDRS,^16^ elliptical partition,^39^,^40^ and quadrantal pRNFL partition^18^,^41^ have been used to examine retinal pathology in different neurodegenerative disorders. The elliptical partition covers a smaller area compared to the ETDRS partition but exclusively includes the horseshoe-like thinning area of the GCIPL as shown in the present study. This explains the higher thickness reduction of the GCIPL in each sector of the elliptical partition and relatively higher differentiation power as the AUCs, compared to the ETDRS partition. On the other hand, the pRNFL scan using the circular scan pattern (equivalent to a linear scan) apparently captured the biggest reduction (i.e., 15.4 μm) of RNFL thickness at the temporal quadrant of the optic nerve head. This may explain how the pRNFL-T had the highest differentiation power, which was even better than the M zone in the present study.

We found that the nasal side of the GCIPL, corresponding to the pRNFL-T, was a vulnerable location for neuroretinal loss in MS. This finding is consistent with previous studies,^18^,^41^ which showed that pRNFL-T is significantly thinner when comparing MS eyes without history of optic neuritis to the control eyes. Previous studies have reported that the reduction in pRNFL-T thickness is associated with optic radiation (OR) damage.^41^,^42^ Lesions within the OR were detected in the majority of patients with MS.^41^ There was a significant correlation between thinning of the pRNFL-T and OR lesion volume,^41^,^42^ indicating that possible retrograding neurodegeneration may play a role in pRNFL and GCIPL thinning.

It is worth noting that an arbitrary partition such as the ETDRS, dedicated to describe the locations of the retinal diseases using fundus photos, may not reflect the pattern of neurologic damage. This may explain why the spatial averaging resulted from the ETDRS partition provided limited performance in the present study. On the other hand, although the ETDRS partition did not provide good differentiation powers, the ETDRS analysis of the GCIPL still provided an overall strong association with EDSS and LCVA when compared to the elliptical partition. The thickness of the GCIPL at the nasal sector (GCIPL-N) was strongly associated with the 1.25% LCVA and was ranked second to the M zone in association with EDSS. This can be explained by the visualized thinning map of the GCIPL, which showed that the nasal quadrant covered the majority portion of the GCIPL thickness reduction area, including the M zone. In contrast, the elliptical partition cut the nasal quadrant in half, which possibly diminished the association between the thickness alterations in these sectors and the clinical manifestations. As such, GCIPL-N may be developed into more sensitive imaging markers for possibly predicting visual function and disease progression.

There are some caveats to the present study. First, we did not do consecutive or random recruitment of our patients from the MS center, which may be a source of bias. This bias would need to be avoided in future studies. Second, the segmentation method using the Orion software and our mapping approach also could be utilized to analyze OCT datasets acquired using commercial OCT systems if the raw dataset is accessible. The UHR-OCT system we used was a custom-made system with sophisticated retinal layer segmentation and partition methods provided by Orion software, and these data may not be directly comparable to previously published data collected with OCT systems that are currently commercially available.^5^,^18^,^26^,^27^ This may limit the generalizability of our current findings at this point. However, further studies using the Orion software along with commercially available OCT devices may validate our findings in this report. Third, we used UHR-OCT to collect the volumetric dataset of the 6 × 6-mm raster scan and analyzed the dataset using the Orion software for mapping and thickness analysis. As the Cirrus HD-OCT datasets of the 6 × 6-mm macular scan and optical nerve head scan were not accessible, we obtained only the Cirrus HD-OCT reports of the GCIPL and pRNFL results. Because the Orion software did not yield thickness results using the elliptical partition and our UHR-OCT did not scan pRNFL, we were not able to directly compare the results.

Nevertheless, we provided the data obtained from the Cirrus HD-OCT and the thickness in the nasal superior and nasal inferior sectors from the elliptical partition of our study cohort. The data from the commercial OCT device may serve as a reference and could be used in the clinic to look at the most profound thinning area. In addition, the Cirrus GCIPL maps in the report also could be a source for searching for localized thickness alteration in the M zone.

Fourth, this is a cross-sectional study and further longitudinal studies with a large sample size may validate these possible image markers. The present study could not provide any evidence that the detailed analysis of the intraretinal layers (i.e., GCIPL thickness in the M zone) could be a more sensitive marker of degeneration in patients with MS over time in longitudinal clinical trials. Fifth, we did not acquire MRI data in this study cohort; therefore, no associations between the eye and brain were examined. Sixth, although the M zone was identified in our MS cohort, the specificity of the M zone to MS will need to be validated in future studies by comparing our current findings to other ophthalmologic (e.g., glaucoma) or neurologic (e.g., dementia) disorders.

In conclusion, the focal thickness reduction of GCIPL was evident and strongly related to visual function and disability in patients with MS without history of optic neuritis. The characteristic thickness alteration pattern may be developed as an imaging biomarker for detecting subtle retinal neurodegeneration and disease progression in MS.
